# Impact of cumulative calorie and protein deficits in critically ill patients

**DOI:** 10.1186/cc9802

**Published:** 2011-03-11

**Authors:** R Dey, M Bhattacharyya, S Todi

**Affiliations:** 1 AMRI Hospitals, Kolkata, India

## Introduction

This study aims to assess the outcome of cumulative protein and calorie deficits in critically ill patients.

## Methods

A prospective observational study conducted in a mixed medical-surgical ICU in a tertiary care hospital in India. Patients receiving nutritional support for 2 days were included. Requirements of calories and protein were fixed as per the ASPEN guidelines. Calorie and protein deficits were calculated daily by subtraction of delivered from prescribed calories and protein in each patient. This deficit (<80% of prescribed were given to the patient) was correlated with outcome and complications.

## Results

A total of 768 patients of age 61 (SD ± 17.67) were analyzed, of which 66.54% were male. In total, 530 (69%) were calorie deficient and 696 (90%) were protein deficient during the whole ICU stay. The correlation coefficient of ICU length of stay (LOS) was -0.443 and -0.465, and of days on mechanical ventilation of alive patients was -0.338 and -0.392 for calorie and protein deficit, respectively (*P *< 0.001). Infectious complications were also significantly correlated (-0.346 for calorie deficit, -0.298 for proteins, *P *< 0.001). The mean calorie deficit of the patients discharged alive from the ICU was -2,135.62 ± 1,918.63, which was less compared with patients who expired (-2,564.44 ± 2,173.45 (*P *= 0.027)). This was also seen in hospital outcome. The mean calorie deficit of patients discharged from hospital was -2,039.36 ± 1,888.82, which was less than the patients who expired after discharge from the ICU (-2,603.99 ± 2,126.53 (*P *= 0.002)). See Tables 1 and 2.

**Table 1:** Correlation of complications with calorie and protein deficits

Variable	Correlation with calorie deficit (*r*)^a ^(*n *= 530)	Correlation with protein deficit (*r*)^a ^(*n *= 696)	*P *value
LOS (ICU)	-0.443	-0.465	< 0.001
Days on mechanical ventilation^b^	-0.338	-0.392	< 0.001
Number of infectious complications	-0.346	-0.298	< 0.001

^a^Correlation coefficient. ^b^Mechanical ventilation days only in alive patients.

**Table 2:** ICU and hospital outcome related to cumulative calorie and protein deficits

	Cumulative calorie deficit	Cumulative protein deficit	*P *value
Alive in ICU	-2,135.62 ± 1,918.63	-258.48 ± 205.58	0.027
Expired in ICU	-2,564.44 ± 2,173.45	-274.44 ± 241.33	0.392
Discharged alive from ICU	-2,039.36 ± 1,888.82	-257.27 ± 208.33	0.002
Expired in hospital	-2,603.99 ± 2,126.53	-271.67 ± 226.85	0.395

Data presented as mean ± SD.

## Conclusions

The cumulative nutrient deficits (calorie and protein) were correlated with increasing number of complications in critically ill patients.

